# Otalgia and Facial Nerve Palsy: Common Symptoms Revealing the Uncommon Pathology of Middle Ear Lymphoma

**DOI:** 10.7759/cureus.25023

**Published:** 2022-05-15

**Authors:** Anna F Jumaat, Hardip Gendeh, Aida W Mohd Mustapha, Geok C Tan, Bee S Goh

**Affiliations:** 1 Otolaryngology - Head and Neck Surgery, Universiti Kebangsaan Malaysia Medical Centre, Kuala Lumpur, MYS; 2 Radiology, Universiti Kebangsaan Malaysia Medical Centre, Kuala Lumpur, MYS; 3 Anatomical Pathology, Universiti Kebangsaan Malaysia Medical Centre, Kuala Lumpur, MYS

**Keywords:** diffuse large b-cell lymphoma, lymphoma, middle ear, otitis media, facial paralysis

## Abstract

Lymphoma of the middle ear and mastoid is uncommon and is rarely diagnosed early. The clinical presentation occurs due to consequences of extensive progressive disease. It can manifest as benign middle ear pathologies such as otitis media; other presentations include facial nerve palsy, hearing loss, and vestibular dysfunction. We treated a case of a 38-year-old male who presented with extranodal involvement of diffuse large B-cell lymphoma (DLBCL) of the middle ear, mastoid, and temporalis muscle, which mimicked an acute otitis media complicated with facial nerve palsy and hearing loss. He has underlying mediastinal and cervical DLBCL diagnosed 20 months before the current presentation. He underwent cortical mastoidectomy and chemotherapy. Despite treatment, he succumbed to the disease. We discuss the clinical significance of middle ear lymphoma by reviewing similar cases in the literature. To conclude, refractory middle ear disease should alert the surgeon of a more sinister underlying pathology in a patient with malignancy.

## Introduction

Lymphomas are classified into Hodgkin’s lymphoma (accounts for 10% of all lymphomas) and non-Hodgkin lymphoma (NHL). NHL can manifest in a broad spectrum of disease, which varies from indolent to the most aggressive malignancies. NHL originates from lymphoid cells, which are at various phases of development. Features of the specific lymphoma subtypes reflect those of the cell of origin. Patients commonly present with lymphadenopathies, but extra-nodal NHL can involve other non-lymphoid tissues or organs. Almost 65% of NHL are diffuse large B-cell lymphoma (DLBCL) together with follicular lymphoma [1]. NHL is commonly encountered during childhood with an incidence of 60% and occurs less frequently among adults. The gastrointestinal tract is the most common site of presentation of NHL, followed by the head and neck region. Involvement of the middle ear and temporal bone is rare, and few cases have been reported in the literature [2-4]. We present a case of extra-nodal DLBCL involving the middle ear and the challenges faced while managing the patient with a literature review on the disease.

## Case presentation

A 38-year-old gentleman with underlying DLBCL of the cervical node presented with sudden onset of right facial paralysis. He was diagnosed with DLBCL germinal center B-cell type (immunohistochemical staining was positive for the cluster of differentiation 10 (CD-10) and B-cell lymphoma 6 (BCL-6), and was negative for multiple myeloma 1 (MUM-1)) 20 months before the current presentation. He had completed eight cycles of R-CHOP (rituximab, cyclophosphamide, hydroxydaunorubicin hydrochloride, vincristine, prednisone) and five cycles of R-MATRix plus (methotrexate, cytarabine, thiotepa, and rituximab). It was preceded by right facial numbness for six months. Subsequently, he developed progressive right facial swelling and diplopia for one month. After that, he developed a one-week history of right facial pain, right-sided non-pulsatile tinnitus, and otalgia. Clinical examination revealed fullness of the right temporal region and right facial palsy with sparing of the forehead, indicating an upper motor neuron palsy. In addition, there was right lateral rectus palsy with paraesthesia of the right side of the face. The pinna and mastoid regions were normal. Otoscopy of the right ear revealed an intact tympanic membrane and no evidence of otitis media. Rinnes test was positive bilaterally, and Webers test was central, indicating symmetrical hearing.

**Figure 1:**
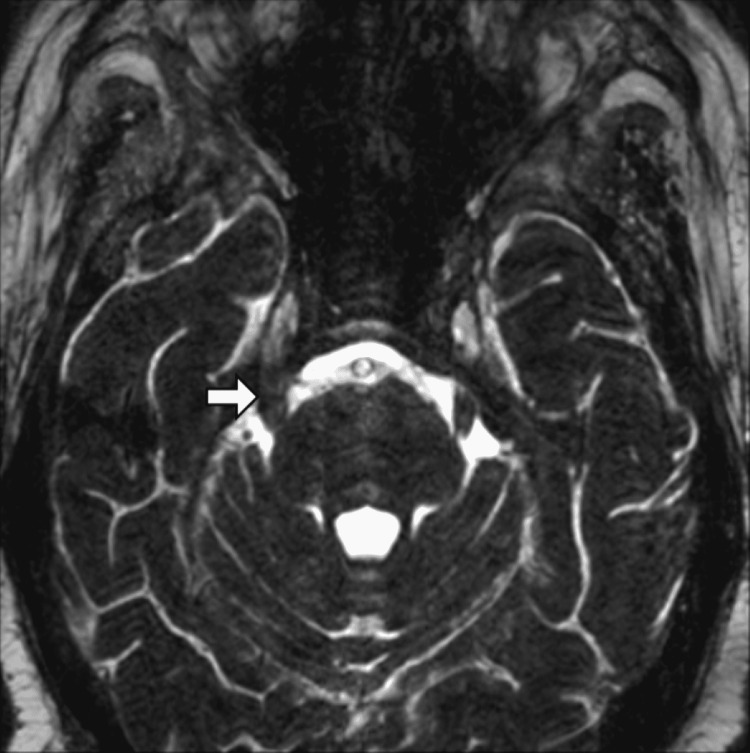
MRI of the brain in axial cut showing enlarged right trigeminal nerve (white arrow). MRI: Magnetic resonance imaging

Previous computed tomography (CT) brain and magnetic resonance imaging (MRI), which was taken five months before the onset of facial paralysis, showed thickening of the right trigeminal nerve, symmetrical focal thickening of the right facial, and vestibulocochlear nerve (Figure 1). However, the middle ear and mastoid were normal. Therefore, he was first diagnosed with cranial nerve palsy (polyneuritis cranialis) secondary to chemo-related leukoencephalopathy for his lateral rectus palsy and facial nerve palsy. 

One month later, he developed an upper respiratory tract infection with right-sided reduced hearing and severe otalgia. There was no worsening of facial paralysis. However, there was an increase in right temporal swelling involving the right mastoid region. Otoscopy examination of the right ear showed that the external auditory canal was edematous. The tympanic membrane was dull and bulging. Pure tone audiometry (PTA) revealed right moderate conductive hearing loss. He was diagnosed with right acute otitis media. Treatment was commenced, and he was prescribed nasal decongestants and antimicrobial ear drops. Despite the initial treatment, the severity of his symptoms intensified. His facial palsy worsened, and the right temporal swelling extended to involve the mastoid region. Intravenous ciprofloxacin was administered given the progression of symptoms.

**Figure 2:**
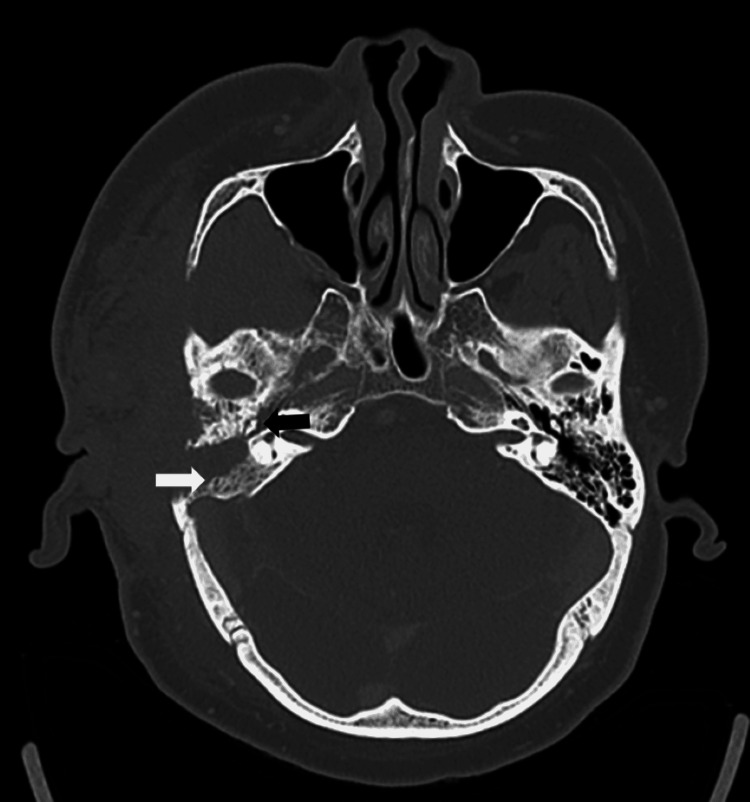
Axial HRCT of the temporal bone showing soft tissue density in right middle ear occupying epitympanum (black arrow). Bony erosion at anterior the wall of right mastoid air cells, fluid-filled right mastoid air cells (white arrow). HRCT: High resolution computed tomography

**Figure 3:**
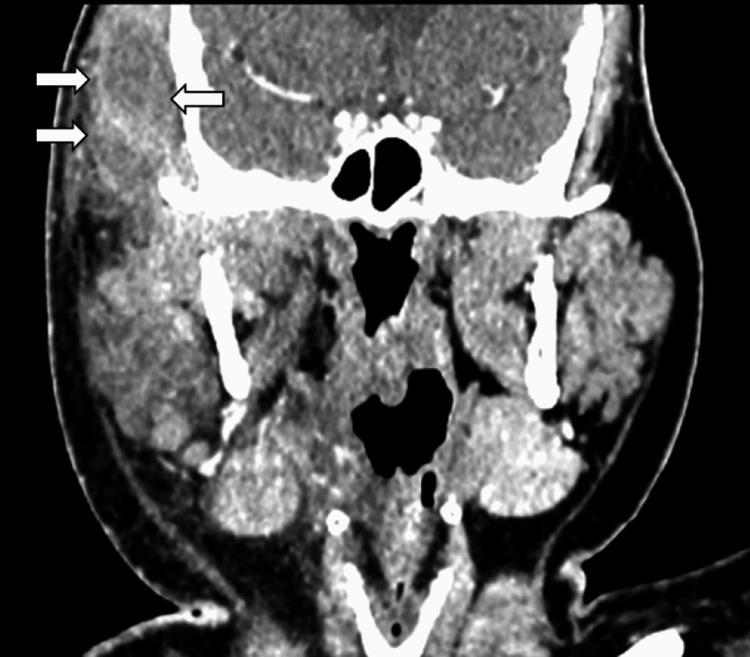
On coronal cut, there is presence of rim-enhancing intramuscular hypodensity within the bulky (white arrows) and thickened right temporalis muscle with enhancement of the right temporalis muscle.

**Figure 4:**
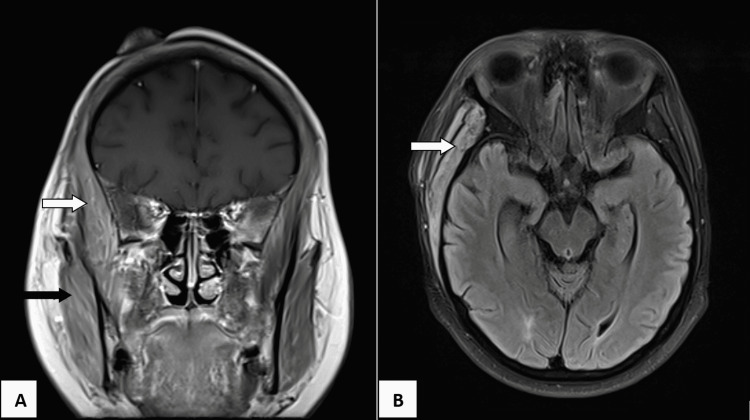
MRI in coronal view (panel A) showing the right temporalis muscle (white arrow) and masseter muscle (black arrow) that are thickened. In axial view (panel B), the right temporalis muscle (white arrow) is thickened with clear plane seen surrounding the muscle. MRI: Magnetic resonance imaging

High-resolution computed tomography (HRCT) images showed non-enhancing soft tissue density occupying the whole right middle ear cavity, with bony erosion at the anterior wall of the right mastoid air cells. There was dehiscence at the tympanic segment of the facial bony canal. The ossicles, scutum, and tegmen tympani were intact (Figure 2). In addition, there was a rim-enhancing intramuscular hypodensity within the right temporalis muscle with an enhancement of the adjacent muscles (Figures 3, 4). He was diagnosed with right acute otitis media, mastoiditis complicated with right temporal abscess, and upper motor neuron facial palsy.

**Figure 5:**
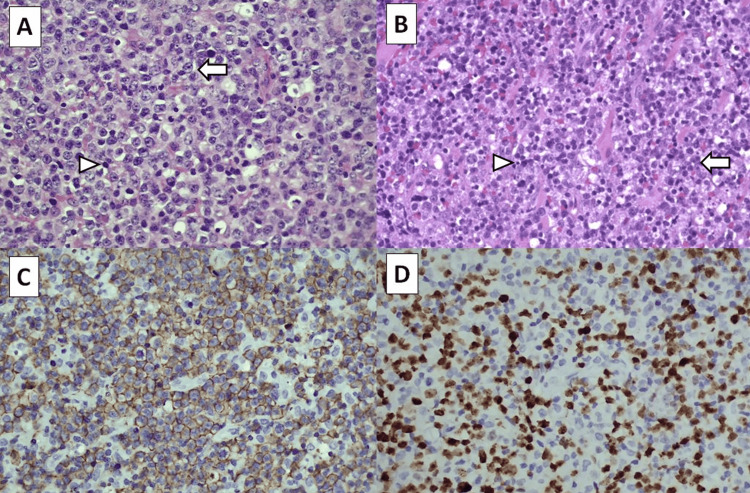
Histology of tumours at the right mastoid (panel A, H&E) and the right temporalis muscle (panel B, H&E) demonstrating malignant lymphoid cells with markedly pleomorphic and enlarged nuclei, small nucleoli and scanty cytoplasm (white arrows). There are many mitoses and apoptotic bodies (white arrow heads). Immunohistochemically, these cells are immunoreactive toward CD20 (panel C) and MUM1 antibodies (panel D). H&E: Hematoxylin & eosin; CD20: Cluster of differentiation 20; MUM1: Multiple myeloma 1

He underwent a right cortical mastoidectomy and myringotomy. Intraoperatively, the middle ear and mastoid were filled with granulation tissue and adherent to the tympanic segment of the facial nerve. The granulation tissue was removed, leaving some remaining tissue at the tympanic segment to avoid further insult and avulsion to the facial nerve. There was no pus present in the temporal region. Histopathological examination of the mass from the right mastoid and temporalis muscle reported DLBCL, activated B-cell subtype (Figures 5A, 5B), with the tumor cells' immunoreactivity toward the cluster of differentiation 20 (CD20) and MUM1 (Figures 5C, 5D). The Ki 67 proliferation index was 80%.

Postoperatively, his headache and otalgia resolved. His hearing had improved, and PTA reported right mild conductive hearing loss. However, he had worsening facial palsy. The histopathological examination results indicated that the patient had relapsed from the disease. Chemotherapy was commenced post-surgery.

The disease progressed despite completing chemotherapy. He developed progressive right-sided hearing loss, worsening facial nerve palsy, and increasing facial and temporal swelling, indicating progression of residual disease. He was counselled for another round of chemotherapy (rituximab, thiotepa, methotrexate) but he refused for any further intervention. Unfortunately, he succumbed to his illness due to advance DLBCL of the cervical lymph node with extra-nodal involvement of the central nervous system, bone marrow, right mastoid, right parotid, and right temporalis muscle.

## Discussion

This is a rare case of NHL DLBCL involving the middle ear, mastoid, and temporalis muscle. His initial presentation with facial palsy, conductive hearing loss, and immunocompromised state mimicked an acute otitis media with mastoiditis. Facial palsy and conductive hearing loss are not usually associated with lymphoma, and as evidenced in this case, lymphoma of the middle ear can be misdiagnosed as a middle ear infection.

Malignancy of the middle ear is rare, and early recognition is mostly futile as the clinical presentation can manifest as other benign pathologies of the middle ear. Differential diagnoses of middle ear neoplasms include squamous cell carcinoma, adenoid cystic carcinoma, adenocarcinoma, basal cell carcinomas, metastatic disease, melanoma, rhabdomyosarcoma, chondrosarcoma, and rarely lymphoma [2].

Lymphomas involving the middle ear can occur due to metastatic foci or direct invasion from nearby locations. It is known that primary lymphomas of the ear are rare [3]. Lymphoma can originate from the mucosa of the middle ear region; the mastoid antrum, tympanum, or the tympanic orifice of the eustachian tube. Deep to the epithelium of the mucosa of the middle ear, there is a layer of lymphoid tissue that can act as the site of origin of primary lymphoma [5]. Another postulation suggests that circulating lymphocytes may ‘home’ in other tissues not considered secondary lymphoid organs [6].

Extranodal involvement may be present in 30% of patients with NHL. The gastrointestinal tract is the most common site of presentation of NHL, followed by the head and neck region [7]. About 85-90% of NHLs are derived from B cells. Many known factors increase the risk of an individual developing NHL, including immune disorders, medicines, infections, lifestyle, genetics, race, family history, and occupational factors. Obesity is a particular risk factor for developing DLBCL. In addition, there are viral and bacterial infections that have been associated with the development of NHL. Hepatitis C and *Coxiella burnetii* infection have been associated with DLBCL [1].

A few challenges were encountered during the management of this particular case. First, the patient presented with typical clinical symptoms of benign middle ear disease, which initially was thought to be attributed to otitis media given his immunocompromised state. However, there was also the acknowledgment of cranial nerve involvement, particularly the facial nerve and trigeminal nerve. The symptoms were a complication due to chemotherapy and neurological involvement of the disease. Therefore, clinicians have to consider possible underlying malignancies for patients who present with intractable otitis media and facial palsy despite adequate antimicrobial therapy. Other warning signs that should alert the clinician are the rapid progress of symptoms and the presence of other associated complications such as hearing loss, vestibular dysfunction, and headache. These may suggest the malignant progress of disease occurring within the middle ear.

Second, despite being on medications, there was intractable otitis media with worsening hearing loss and increasing temporal and mastoid fullness, evident by clinical examination and radiological findings. The etiology of infection was a concern based on the patient’s clinical signs and symptoms. Although the initial symptoms of this type of NHL resemble those of a middle ear disease with probable abscess formation of the temporal region, antibiotics seem not to help resolve these symptoms.

Third, the main priority was to treat the patient symptomatically by restoring ventilation to the middle ear and mastoid region and draining an abscess if encountered during surgery. Obtaining tissue for diagnostic purposes was necessary for diagnosis confirmation and determining an appropriate treatment strategy.

Diagnosis is based on an adequate biopsy sample, which a well-trained haemato-pathologist should review. It has been advised that the sample should preferably be obtained from an excisional biopsy of a lymph node or tissue from a solid tumor. Procuring sufficient tissue for immunohistochemical and genetic studies will enable the pathologist to reach the correct diagnosis [1].

Lymphomas of the middle ear are sporadic, the prognosis of this disease is unknown, and its treatment has not been clearly established. The R-CHOP regimen includes cyclophosphamide, doxorubicin, vincristine, prednisone, and rituximab administered every three weeks as the standard treatment for DLBCL. However, there are studies that indicate that more intensive regimens might improve the outcome in young patients, such as ACVBP-R (doxorubicin, cyclophosphamide, vindesine, bleomycin, prednisone, and rituximab) and EPOCH-R (etoposide, prednisone, vincristine, cyclophosphamide, doxorubicin, and rituximab) infusional chemotherapy regimen [8,9].

Poor prognosis is observed in patients with DLBCL who relapse after achieving complete remission or in patients with inadequate response to the initial regimen. An autologous hemopoietic stem-cell transplant is an option for treatment if patients respond to a salvage chemotherapy regimen [1]. Though the mainstay of treatment in lymphoma is radiotherapy and chemotherapy, surgery may be indicated to restore ventilation of the middle ear, debulking of tumor, tissue biopsy, and alleviate symptoms associated with disease spread.

Lymphoma of the middle ear, specifically NHL DLBCL of the middle ear, is rare. A review of the literature was performed using Ovid Medline and Web of Science (WoS) search engines. Search results revealed eight case reports of patients with malignancy involving the middle ear (Table 1).

**Table 1:** Literature review of DLBCL of the middle ear. ALL; Acute lymphocytic leukemia

Author	Age	Sex	Presentation	Surgery	Treatment	Outcome
Ding et al 2019 [7]	20	Male	Conductive hearing loss, otalgia, aural fullness, primary ALL	Excisional biopsy	Chemotherapy	Died; Secondary
Alquanee 2018 [2]	62	Male	Otalgia, otorrhoea, facial palsy	Cortical mastoidectomy, facial nerve decompression, middle ear exploration	Chemotherapy	Alive; Facial palsy; Primary
Siddiahgari 2016 [4]	2	Male	Otalgia, otorrhoea, facial palsy	Mastoidectomy	Chemotherapy	Alive; Primary
Ryou et al 2015 [10]	25	Male	Hearing loss, otalgia, facial palsy, vertigo	Lumbar puncture with cerebrospinal fluid cytology	Chemotherapy, intrathecal methotrexate	Dead; Secondary
Merkus et al 2000 [11]	75	Male	Otalgia, otorrhoea, facial palsy	Biopsy	Chemotherapy, radiotherapy, intrathecal methotrexate	Alive; Primary
Tucci et al 1992 [12]	5	Male	Otalgia, otorrhoea, facial palsy, conductive hearing loss	Biopsy of middle ear tissue	Chemotherapy	Resolved facial palsy
Tucci et al 1992 [12]	66	Male	Sensorineural hearing loss, vertigo	Mastoidectomy	Chemotherapy, radiotherapy	Dead
Toriumi et al 1988 [13]	76	Male	Sensorineural hearing loss, facial palsy	Cortical mastoidectomy with facial nerve decompression	Unknown	Unknown

The first case to describe NHL DLBCL of the middle ear was by authors Toriumi et al. [13] in 1988. Our review shows that a wide variety of lymphoma subtypes can occur in the middle ear and can be either primary or secondary malignancy. Most patients diagnosed with NHL DLBCL of the middle ear are male adults, with one case reported in a two-year-old [4]. It is also observed that most patients present with symptoms of otalgia, otorrhoea, hearing loss, and facial palsy. Unfortunately, these clinical presentations are also present in benign ear diseases and can lead the physician to misdiagnosis.

## Conclusions

DLBCL occurring at extranodal sites can present with unique clinical features depending on the anatomical region of involvement. Hence, arriving at the final diagnosis of malignancy of the middle ear is rarely made early. This is due to the distinctive anatomy of the middle ear and clinical presentation, which can mimic benign and common ear pathologies. This, in turn, delays the correct diagnosis of malignancy, further disrupting the treatment. Imaging and histopathology investigations are crucial in determining the correct pathology and, at times, encompass surgical intervention. Malignant lymphoma should be considered a differential diagnosis in cases of therapy-resistant ear disease with progression.
